# Effect of anesthesia on the success rate of external cephalic version: GRADE- assessed systematic review and meta-analysis of randomized controlled trials

**DOI:** 10.1186/s13643-024-02616-y

**Published:** 2024-07-30

**Authors:** Liming Lei, Zhiyong Fang, Chenyang Xu, Zhaohui Wang, Hui Li, Li Ma

**Affiliations:** 1 Department of Anesthesiology, Women’s Hospital of Nanjing Medical University, Nanjing Women and Children’s Healthcare Hospital, 123 Tianfei Lane, Nanjing, 210004 China; 2 Department of Anesthesiology, Lishui People’s Hospital, Nanjing, Jiangsu 211200 China

**Keywords:** Breech presentation, Anesthesia, External cephalic version, Vaginal delivery, Cesarean delivery

## Abstract

**Background:**

External cephalic version (ECV) is a medical procedure in which an extracorporeal manipulation is performed to render the breech presentation (BP) fetus in the cephalic position. The use of anesthesia to facilitate repositioning has been evaluated in various randomized clinical trials (RCTs), but its potential effectiveness remains controversial.

**Methods:**

A systematic literature search was carried out in 8 electronic databases. In the meta-analysis, a random effects model was used to calculate the pooled relative risk (RR) and its 95% confidence interval (CI), and the pooled standardized mean difference (SMD) and its 95% CI, in order to systematically assess the effect of anesthesia on the success rates of ECV, vaginal delivery, cesarean delivery as well as other outcomes. Relevant subgroup analyses, publication bias test and sensitivity analyses were also conducted.

**Results:**

This review included 17 RCTs. Women who received anesthesia had a significantly higher incidence of successful ECV (RR: 1.37, 95% CIs: 1.19-1.58) and vaginal delivery (RR: 1.23, 95% CIs: 1.03-1.47), and a significantly lower incidence of cesarean delivery (RR: 0.69, 95% CIs: 0.53-0.91), compared with those who did not.

**Conclusion:**

The administration of anesthesia not only significantly reduces maternal pain but also significantly increases the success rate of ECV in women with malpresentation at term, leading to a significant rise in the incidence of vaginal delivery. However, it may increase the incidence of maternal hypotension.

**Systematic review registration:**

The protocol was prospectively registered with PROSPERO, registration CRD42022381552.

**Supplementary Information:**

The online version contains supplementary material available at 10.1186/s13643-024-02616-y.

## Introduction

The breech position (BP) is a prevalent anomalous fetal position encountered in clinical settings. It is noteworthy that roughly 96% of BP fetuses spontaneously reorient themselves to the cephalic position by 32 weeks of gestation. However, the remaining 4% of fetuses keep in the BP until full-term delivery [1]. The breech vaginal delivery poses a significant risk of cord prolapse, trauma, asphyxia, and even death of the newborn. As a result, many medical institutions have made cesarean delivery the standard approach for BP [2]. However, cesarean delivery has a number of possible adverse effects on the mother and newborn, including abnormal uterine bleeding, maternal urinary difficulties, neonatal asthma, and childhood overweight and obesity, which are gradually gaining public attention [3, 4]. Fortunately, the external cephalic version (ECV) provides a potential alternative to cesarean delivery, and its success rate is high in reducing the cesarean rate and overall cost of care without increasing the risk of perinatal complications [2, 5]. Moreover, ECV has been recommended by the Obstetrics and Gynecology societies of the United Kingdom, the United States, and other countries [6, 7].

ECV is a medical procedure in which the BP fetus is externally manipulated to present in the cephalic position. Its success rate ranges from 35% to 86%, with an average success rate of 58% [8]. Gestational age, number of deliveries, placental position, expertise of the obstetrician, etc. may affect the success rate of ECV [9]. And the reaction of pregnant women to the pain produced by the ECV procedure largely influenced the procedure. In the early stages, many pregnant women ask to stop the procedure midway because of the unbearable pain [9]. With the development of medical technology, various anesthetic interventions have been used to assist in the implementation of ECV, such as epidural anesthesia [8], spinal anesthesia [10], inhaled anesthesia [11], intravenous anesthesia [12] and so on.

However, the use of anesthesia in ECV is controversial. While some studies confirmed that anesthetic analgesia significantly enhances the success rate of ECV [13, 14], others demonstrated no difference in the success rate of ECV with or without anesthesia [11, 15]. In addition, the toxic effects of anesthetics and excessive external force may cause adverse events [16, 17]. Assessing the possible risks and benefits of using anesthesia during ECV can help physicians and the public make benefit-maximizing decisions, which has important practical implications. A systematic review and meta-analysis by Goetzinger et al. retrieved randomized controlled trials (RCTs) published from 1966 to April 2011 and found that regional anesthesia was associated with higher ECV success rates and did not increase the incidence of adverse events [18]. Magro-Malosso et al. performed a pooled analysis of RCTs through January 2016 and evaluated the effect of neuraxial analgesia on ECV compared to intravenous anesthesia or no anesthesia. And this meta-analysis concluded that neuraxial analgesia improves the success rate of ECV [19]. In recent years, there has been an increasing number of RCTs on the effects of anesthesia on ECV. The aim of our study was to pool relevant published RCTs to systematically assess the effect of anesthesia (general and regional anesthesia) on the success rate of ECV and possible side effects.

## Methods

Our study has been registered (registration number: CRD42022381552) with the PROSPERO database before December 16, 2022 (https://www.crd.york.ac.uk/prospero/display_record.php?ID=CRD42022381552). We did not use individual data but published data. These data have been widely utilized in research and are generally available. Therefore, we confirm that any aspect of the work covered in this manuscript has been conducted with ethical approval. We used the Cochrane Handbook for Systematic Reviews of Interventions for the preparation and conduct of this meta-analysis. We reported this meta-analysis with reference to Preferred Reporting Items for Systematic Review and Meta-Analyses (PRISMA) guidelines [20].

### Search strategies

The literature searching was completed on January 2, 2024 for relevant available articles (no restrictions on language or date of publication) from the following databases: (1) PubMed; (2) Embase; (3) Cochrane library; (4) Web of Science; (5) Sinomed (CBM); (6) China National Knowledge Infrastructure (CNKI); (7) Wanfang Data Knowledge Service Platform; (8) China Science and Technology Journal VIP Database. The registration search was completed before January 2, 2024 and the relevant data were retrieved from the following registration pools: (1) ClinicalTrials.gov; (2) International Clinical Trials Registry Platform (ICTRP); (3) The EU Clinical Trials Register; (4) Chinese Clinical Trial Registry. The relevant retrieval strategy of different databases was shown in Table s1. Relevant Chinese technical terms for the Chinese databases were used to search for published articles. Furthermore, references of all relevant articles and reviews were retrieved to search for additional eligible studies. The primary outcome was successful ECV, incidence of vaginal delivery, and cesarean delivery. Secondary outcomes were incidence of vaginal breech delivery, emergency cesarean delivery, transient bradycardia, nonreassuring fetal testing (excluding transient bradycardia) after ECV, maternal discomfort, maternal pain score, abruption placentae, vaginal bleeding or postpartum hemorrhage, satisfaction scores, and hypotension. More information on the time points and scales specifics of the different studies outcomes was shown in Table s2.

### Inclusion and exclusion criteria

#### Inclusion criteria

Studies were included in this meta-analysis if they met the following criteria: (1) research must be the RCT study; (2) The study population was only women ≥36 weeks of gestation; (3) The exposure factors were general or regional anesthesia, including epidural anesthesia, spinal anesthesia, inhalation anesthesia and intravenous anesthesia; (4) The control groups were all blank controls without anesthesia (placebo); (5) The data of the experimental and control groups were complete, including the number of occurrences in the experimental group and the total number of occurrences in the control group and the total number of occurrences in the control group; (6) If the same population had multiple publications, the study with the larger sample size or more information available was selected.

#### Exclusion criteria

The exclusion criteria were as follows: (1) non-RCTs studies; (2) studies in women <36 weeks' gestation; (3) unrelated, repeatedly published literature with no relevant data; (4) comments or letters to the editor, case reports, and abstracts only; and (5) preprint servers, such as medRxiv/bioRxiv, etc.

#### Data extraction

After removing duplicates, all abstracts and titles were independently screened by two evaluators (LL and ZF) to remove irrelevant articles. We downloaded and read the full text of potential studies and included those that met the selection criteria for this systematic review. Data extraction of the included literature was performed by 2 investigators (LL and ZF). Data extraction included: name of first author, year of study, study region, number of study subjects, type of anesthesia, primary outcome and secondary outcomes. Corresponding authors were contacted twice (reminder emails were sent after the first email) if the data required for the meta-analysis were not available in the published article.

#### Quality assessment

Two reviewers independently assessed the risk of bias within each study by using a Cochrane risk of bias instrument [21, 22]. We evaluated random sequence generation, allocation concealment, blinding of participants and personnel, blinding of outcome assessment, incomplete outcome data, selective outcome reporting and other sources of bias. The assessors resolve the differences through discussion, and two arbitrators decide on any unresolved differences. When risk of bias varied across included studies, we stratified studies according to risk of bias and produced two estimates of the intervention effect: from trials at low risk of bias and from all studies. The Grading of Recommendations Assessment, Development and Evaluation (GRADE) framework was used to determine the certainty of the evidence [23].

### Statistical analysis

Statistical analyses of all data were performed with Stata (version 15.0; Stata Corp, College Station, TX) and RevMan (version 5.3; Cochrane library) software. Relative risk (RR) is the frequency of the outcome in the exposed group divided by the frequency of the outcome in the control group. RR and 95% confidence intervals (95% CIs) were estimated for binary outcomes. Standardized mean difference (SMD) was obtained by dividing the difference between the estimated means of the two groups by the mean standard deviation[24]. The SMD express the size of the treatment effect in each study relative to the variability observed in that study[25] . SMD and 95% CIs were calculated for continuous outcomes, which eliminates the effect of dimension when combining statistics[26]. The pooled RR is considered statistically significant if 95% CI did not contain 1, and the pooled SMD is considered statistically significant if 95% CI did not contain 0. To generalize our findings beyond the included studies, the random-effects model is the most appropriate model for meta-analysis[27]. Individual and pooled estimates were illustrated using forest plots. Subgroup analyses were also performed to examine the impact of anesthesia on the success rate of ECV according to study region and type of anesthesia. Sensitivity analyses were performed to explore whether a study had a substantial effect on the outcome. Publication bias was assessed qualitatively by funnel plots and quantitatively by Begg test and Egger's test. In all analyses, P values less than 0.05 were considered statistically significant.

## Results

### Study selection and characteristics

**Fig. 1:**
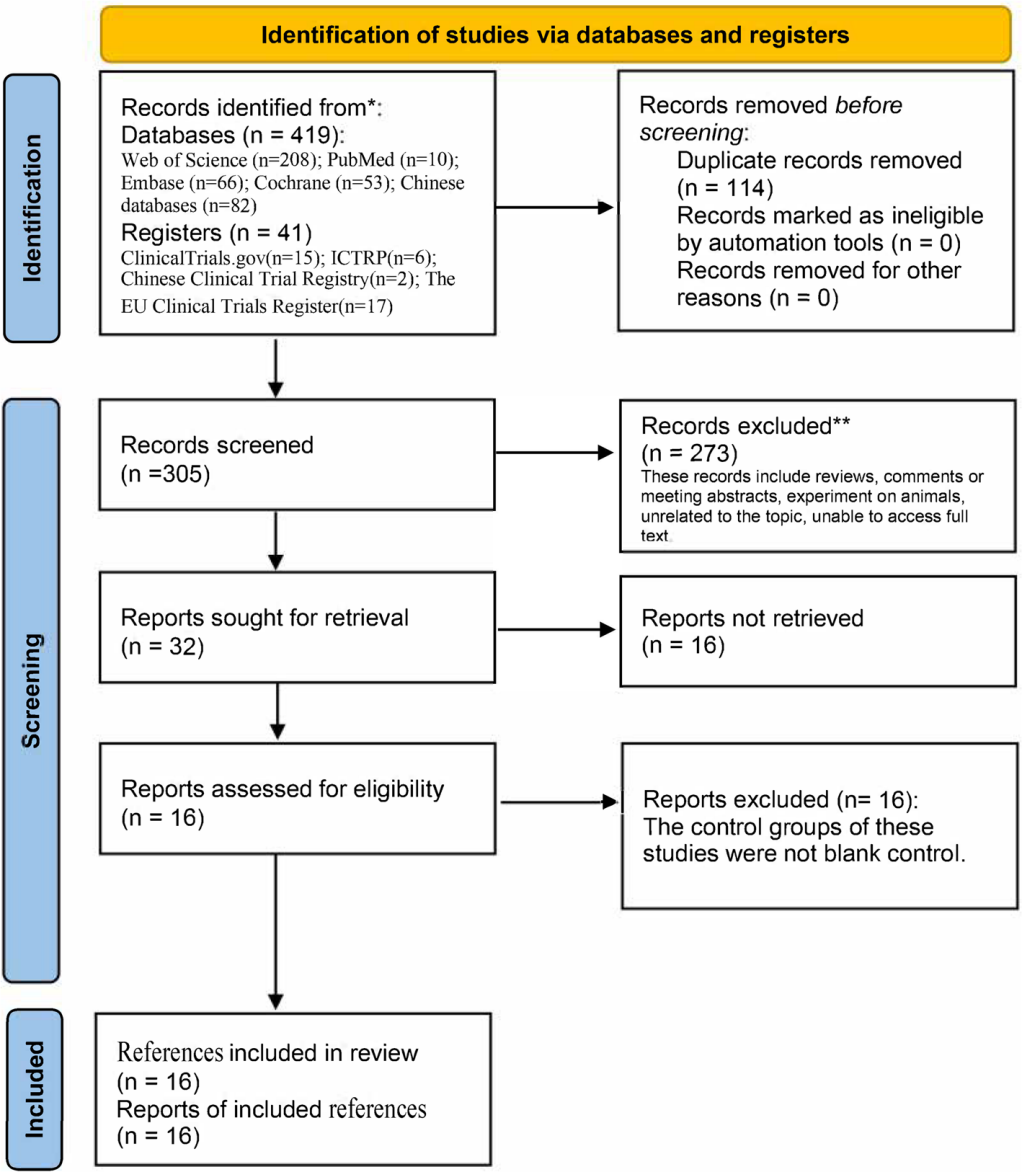
Flow diagram of the study search and selection process

An electronic search finalized 419 publications, and 32 potentially relevant studies were finally screened after eliminating duplicates and screening titles and abstracts. Of the 32 potentially relevant studies, a total of 16 publications met the inclusion criteria [8–12, 14, 15, 28–36]. The detailed process of literature screening is shown in Fig. 1. Therefore, 16 publications with a total of 17 RCTs were finally included in this meta-analysis, of which the study by Khaw et al. [10] included two RCTs . The characteristics of the included studies are shown in Table 1. The inclusion and exclusion criteria were presented in Table 2.

**Table 1:** Characteristics of the included trials

**Study**	**Study location**	**Sample size (anesthesia/control)**	**Maternal age (anesthesia vs control)**	**Analgesia type**	**Control group**	**Primary outcome**	**Other outcomes**
Schorr et al, 1997 [27]	Mississippi, USA	69 (35/34)	27.7±6.1 vs 25.8±6.6	Epidural: 2% lidocaine with 1:200,000 epinephrine	No anesthetic intervention	Successful ECV	Patient discomfort, epidural complication, discharge (after ECV), reverted to breech, cesarean delivery, vaginal delivery, hospital stays.
Dugoff et al, 1999 [9]	Colorado, USA	102 (50/52)	24.3±0.9 vs 26.8±0.9	Spinal: 10 mg sufentanil and 1 mL of 0.25% bupivacaine	No anesthetic intervention	Successful ECV	Vaginal delivery rate, cesarean delivery rate, transient bradycardia, hypotension, abruption, patient discomfort.
Mancuso et al, 2000 [8]	Hawaii, USA	108 (54/54)	28.5±4.8 vs 28.2± 4.8	Epidural: 2% lidocaine with 1:200,000 epinephrine and 100 ug of fentanyl	No anesthetic intervention	Successful ECV	Vaginal delivery rate, cesarean delivery rate, transient bradycardia, cephalic presentation after version attempt, cephalic presentation at delivery.
Weiniger et al, 2007 [28]	Israel	70 (36/34)	24.6±3.8 vs 28.1±4.1	Spinal:7.5 mg bupivacaine	No anesthetic intervention	Successful ECV	Uterine tone relaxed, easy palpation of fetal head, further analgesia offered for ECV due to pain, VAS pain score (0–10).
Weiniger et al, 2010 [29]	Israel	64 (31/33)	28.5[21,–40] vs 28.6[20,–36]	Spinal: 7.5 mg bupivacaine	No anesthetic intervention	Successful ECV	Vaginal delivery rate, cesarean delivery rate, transient bradycardia before/after ECV, uterine tone relaxed, easy palpation of fetal head, V AS pain score (0 –10), maternal hypotension.
Burgos et al, 2013 [11]	Biscay, Spain	450 (300/150)	In both cohorts, the mean age was 33 years.	Inhaled N_2_O in a 50:50 mix with oxygen	No anesthetic intervention	Successful ECV	Emergency cesarean section rate after ECV, complications, cesarean delivery, umbilical cord pH < 7.00, rate of admission to neonatal unit, median and interquartile range of pain score after the ECV.
Munoz et al, 2014 [12]	Spain	60 (31/29)	32.9±4.9 vs 32.5±5.7	Intravenous injection: 100 ml remifentanil 0.01 mg/ml	Saline placebo	Maternal pain	Number of PCA demands, successful ECV, vaginal delivery rate, cesarean delivery rate, transient bradycardia, adverse effects.
Khaw et al, 2015 [10]	China	three groups: 189 (63/63/63)	group 1 vs group 2 vs group 3: 32[23–42] vs 32[23–42] vs 31[20–39]	Group 1: Spinal, 9 mg bupivacaine and 15 ug remifentanil Group 2: Intravenous anesthesia	No anesthetic intervention	Successful ECV	No of ECV attempts, duration of ECV, Vaginal delivery rate, cesarean delivery rate, visual Pain Score (0–100), visual Sedation Score (0–100).
Liu et al, 2016 [14]	China	152(76/76)	34.1±4.2 vs 33.8±3.9	Intravenous injection: paracetamol 1 g in 100 mL saline + remifentanil (infused at 0.1 ug/kg/min + demand boluses of 0.1 ug/kg)	Saline placebo	Maternal pain	Successful ECV, vaginal delivery rate, cesarean delivery rate, transient bradycardia.
Wang et al, 2017 [30]	China	144(72/72)	33.2±4.6) vs 32.9±5.1	Intravenous paracetamol 1 g in 100 mL saline 5 minutes before ECV. Subsequently, they received a patient-controlled analgesia at 0.1 ug/kg/min for 3 minutes and then rescue boluses on demand of 0.1 ug/kg and a lockout period of 4 minutes.	Saline placebo	Maternal pain	Successful ECV, vaginal delivery rate, cesarean delivery rate, transient bradycardia, number of PCA demands, satisfaction score, adverse events.
Yang et al, 2019 [31]	China	46 (25/21)	27.15±2.73 vs 25.48±3.67	Epidural anesthesia	No anesthetic intervention	Successful ECV	Vaginal delivery rate, cesarean delivery rate, placental abruption, postpartum hemorrhage neonatal asphyxia (mild).
Dochez et al, 2020 [15]	France	150(74/76)	32.9±5.6 vs 31.0± 4.6	Inhaled nitrous oxide in a 50:50 mix with oxygen	Medical air	Successful ECV	Vaginal delivery rate, cesarean delivery rate, transient bradycardia, degree of pain with VAS, emergency cesarean before labor, emergency cesarean in labor, admission to neonatalology unit.
Zhang et al, 2020 [32]	China	80(40/40)	28.91±2.90 vs 28.76±2.88	Spinal anesthesia: receive low concentration of ropivacaine combined with low dose fentanyl, where the concentration of ropivacaine is 0.25% and the dose of fentanyl is 15 g.	No anesthetic intervention	Successful ECV	cesarean delivery rate
Han et al, 2020 [33]	China	104 (63/41)	28.3±3.1 vs 28.2±3.0	Spinal anesthesia: low concentration ropivacaine (0.25%) combined with low dose fentanyl (15 ug)	No anesthetic intervention	Successful ECV	Vaginal delivery rate, cesarean delivery rate, number of cases of obstructed labor and stillbirth, premature rupture of membranes, postpartum hemorrhage, neonatal asphyxia, premature birth, umbilical cord wrap, neonatal birth injuries, maternal pain.
Straube et al, 2021 [34]	North Carolina	48 (23/25)	32.5±4.7 vs 31.8±4.3	Inhaled 50:50 mixture of N_2_O and oxygen	100% oxygen	Maternal pain	Successful ECV, cesarean delivery rate, anxiety, satisfaction, eventual delivery mode, procedural difficulty, and ECV complication rates.
Yang et al, 2023 [35]	China	201 (67/134)	30.04 ± 4.22 vs 31.22 ± 4.18	Injection of 0.25 mg subcutaneous terbutaline and 7.5 mg of intrathecal ropivacaine in the left side lying position.	No anesthetic intervention	Successful ECV	Clinical outcomes, such as placenta abruption, cord prolapse, hemorrhage and Apgar score.

*USA* the United States, *ECV* External cephalic version, *N*_*2*_*O* Nitrous oxide, *VAS* Visual analog scale, *PCA* Paracetamol

**Table 2:** Inclusion and exclusion criteria of the included trials

**Study**	**Inclusion criteria**	**Exclusion criteria**
Schorret al, 1997 [27]	GA >37 wks	Patient exclusion factors were placenta previa, evidence of fetal compromise, intrauterine growth restriction, and rupture of membranes.
Dugoff et al, 1999 [9]	GA ≥36 wks, breech presentation (not transverse or oblique lie); reactive NST; intact membranes with a minimum 2 ×2 cm pocket of amniotic fluid	Gross fetal anomalies, uterine malformation, EFW >4000 g, IUGR, placenta previa; known maternal history of third-trimester vaginal bleeding; labor, no contraindications to spinal anesthesia or terbutaline
Mancuso et al, 2000 [8]	At least 18 y with singleton pregnancies of at least 37 wks in breech or transverse presentations, intact membranes, EFW between 2000 and 4000 g, reassuring FHR testing	Placenta previa, prior classical cesarean delivery, third-trimester bleeding, AFI <5 cm or >25 cm, known uterine malformation, uncontrolled hypertension, suspected major fetal anomaly, active-phase labor
Weiniger et al, 2007 [28]	ASA status I-II, GA 37e40 wks, no fetal abnormality	Previous uterine surgery or uterine anomaly, contraindication for vaginal delivery and for regional analgesia, patient refusal of regional analgesia, neuropathy, severe back pain with neurological radiation, poor communication, BMI ≥ 40 kg/m^2^
Weiniger et al, 2010 [29]	ASA status I-II, GA 37-40 wks, no fetal abnormality (including IUGR), no contraindication for vaginal delivery and for regional analgesia	Previous cesarean delivery, previous myomectomy with uterine cavity penetration or uterine anomaly, BMI ≥40 kg/m^2^, AFI <7 cm, neuropathy, severe back pain with radicular radiation, request for elective cesarean delivery
Burgos et al, 2013 [11]	GA ≥37 weeks, singleton pregnancy, informed consent obtained	ECV were placenta previa, placental abruption, uterine malformation, oligohydramnios, signs of fetal distress, fetal death, severe fetal malformations, multiple pregnancy, Rh incompatibility, clotting disorders, and any indications for cesarean section, contraindication to surgery or N_2_O use.
Munoz et al, 2014 [12]	36–41 weeks of gestation with a non-cephalic presentation confirmed by ultrasound scan	Fetal abnormalities, intrauterine fetal death, suspicion of fetal growth restriction, fetal weight above 3800 g, maternal cardiovascular disease, American Society of Anesthesiologists class >2, severe hypertension, allergy to any trial medications, amniotic fluid index<4 cm, Doppler cerebroplacental ratio >5th percentile, abnormal cardiotocographic recordings, contraindications to vaginal delivery, uterine abnormalities, coagulation disorders, Rhesus incompatibility, multiple gestation, rupture of membranes and/or placental abruption
Khaw et al, 2015 [10]	American Society of Anesthetists status I-II, term parturients, breech-presenting fetus	Contraindications to ECV including patients with known uterine scar or anomaly, unexplained third-trimester bleeding, obstetric or medical conditions complicating pregnancy, compromised fetus, nuchal cord, fetal anomaly, premature rupture of membranes, labor
Liu et al, 2016 [14]	The study population consisted of singleton pregnancies with breech presentation at term (≥37 weeks), confirmed by ultrasound	History of prior uterine surgery, uterine abnormalities, multiple pregnancy, contraindications to vaginal delivery, maternal cardiovascular disease, severe hypertension, American Society of Anesthesiologists class >2, allergy to the trial medications, prelabor ruptured membranes, placental abruption, fetal anomaly, intrauterine fetal death, and fetal weight above 3800 g. In addition, participants who received ECV, and also the moxibustion therapy, to correct the breech presentationat before the study recruitment were also excluded
Wang et al, 2017 [30]	Nulliparous women with singleton BP at term (≥37+0 weeks), and the eligibility of all subjects was confirmed with ultrasound examination	Subjects were excluded in the presence of fetal abnormalities, intrauterine fetal death, multiple pregnancy, prior uterine surgery, maternal cardiovascular disease, severe hypertension, fetal weight >3800 g, American Society of Anesthesiologists class >2, allergy to remifentanil and its placebo, ruptured membranes, and placental abruption
Yang et al, 2019 [31]	Full term (≥37 weeks), singleton, breech	History of scarred uterus, abnormal placental position, uterine anomalies, severe comorbidities or complications, and contraindications to vaginal delivery
Dochez et al, 2020 [15]	GA of at least 36 weeks and scheduled for external cephalic version	Maternal age younger than 18 years, relative contraindications for ECV (contraindication to vaginal delivery, placenta previa, intrauterine fetal death, oligohydramnios, fetal heart rate abnormalities or maternal HIV seropositivity), and contraindications linked to the equimolar mixture of N_2_O and oxygen (pneumothorax, ophthalmic gases [SF6, C3F8, or C2F6] in the 3 months before ECV, alteration of consciousness, or need for continuous oxygen therapy for a pulmonary illness)
Zhang et al, 2020 [32]	Singleton pregnancy, no umbilical cord entanglement by ultrasound, fetal position is breech	Contraindications to intralesional anesthesia such as abnormal intracranial pressure, BMI over 40 kg/m^2^, gestational diabetes mellitus, gestational hypertension and other gestational syndromes; multiple pregnancies
Han et al, 2020 [33]	Primigravida with singleton pregnancy, gestational cycle of (37±2) weeks, fetal position in breech position diagnosed by ultrasound, no cord entanglement, familiar with and aware of the whole experimental study, and voluntarily enrolled	Patients with multiple pregnancies or trans-pregnant women, pregnant women with gestational hypertension, diabetes mellitus and other gestational syndromes, patients with BMI>40 kg/m^2^ and patients with intracranial pressure abnormalities and other contraindications to intravertebral anesthesia are excluded
Straube et al, 2021 [34]	At least 18 years old, 37-weeks' gestation or beyond, singleton pregnancy, breech presentation, and American Society of Anesthesiology physical status I-III	Non-English speaking, patient refusal, contraindication to N_2_O use (recent eye or brain surgery or pneumothorax), and patients who were receiving NA for ECV
Yang et al, 2023 [35]	Singleton pregnancy; a noncephalic presentation at 36–38 weeks gestation and scheduled for ECV	Cesarean delivery cannot be avoided, diagnosed with placenta previa or twin pregnancy; contraindications were early labor, oligohydramnios or rupture of membranes, severe fetal growth restriction, uterine malformation, prior abruption, and prior cesarean delivery; incomplete history and surgery records were available; and participants requested repeat cesarean delivery during delivery.

*GA* Gestational age, *wks* weeks, *NST* Nonstress test, *EFW* Estimated fetal weight, *IUGR* Intrauterine growth restriction, *FHR* Fetal heart rate, *AFI* Amniotic fluid index, *ASA* American Society of Anesthetists, *BMI* Body mass index, *ECV* External cephalic version, *N*_*2*_*O* Nitrous oxide, *BP* Breech presentation, *HIV* Human immunodeficiency virus

### Synthesis of results

**Fig. 2:**
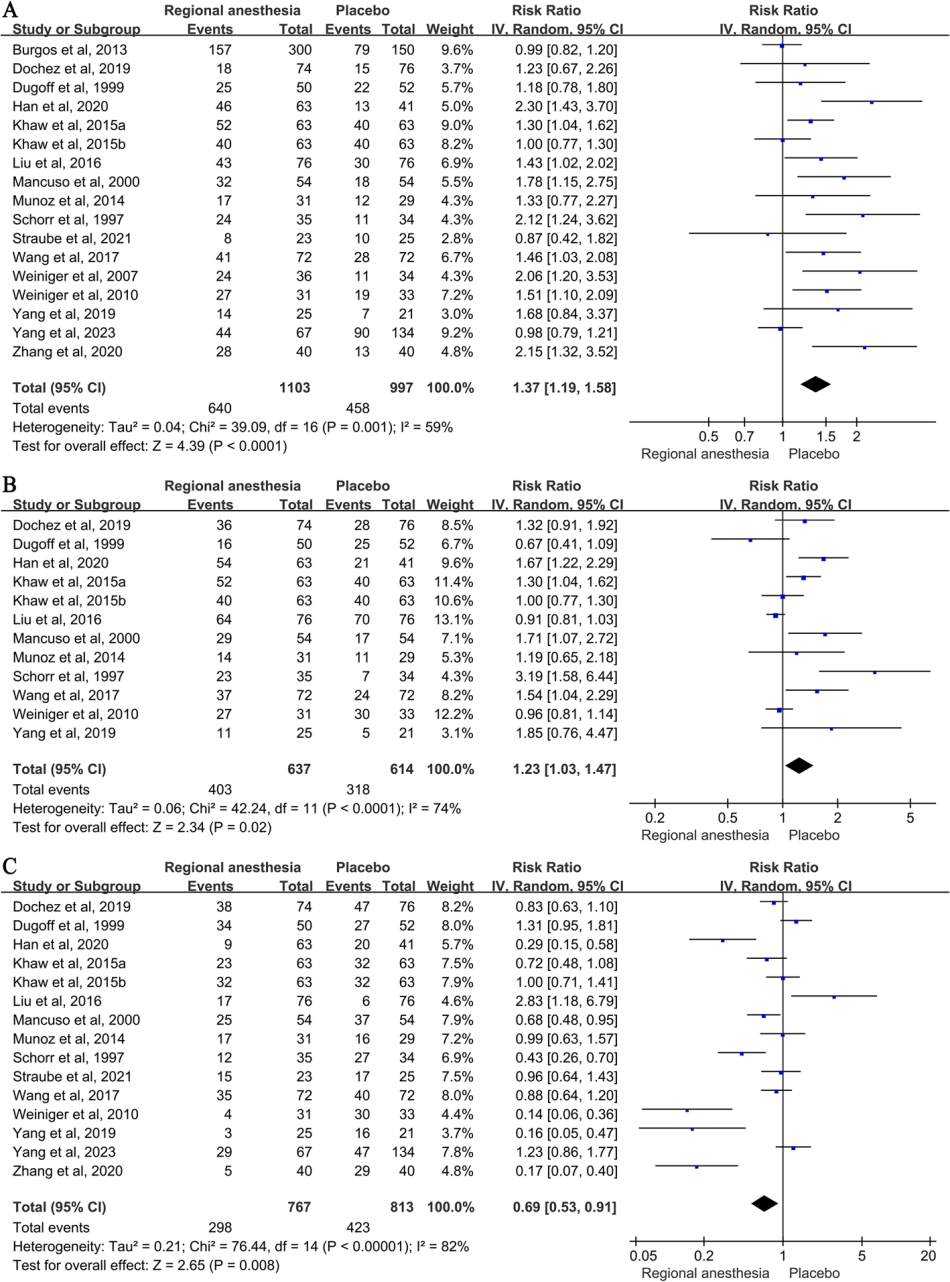
Results of meta-analysis of anesthesia on the risk of successful ECV, vaginal delivery (B) and cesarean delivery (C)

Table 3 shows the pooled data of the primary and the secondary outcomes of the meta-analysis. Women who received anesthesia had a significantly higher incidence of successful ECV (58.2% vs 45.9%; RR: 1.37, 95% CIs: 1.19-1.58, *P*=0.001, *I*^*2*^=59%) and vaginal delivery (63.3% vs 51.8%; RR: 1.23, 95% CIs: 1.03-1.47, *P*=0.02, *I*^*2*^=74%), and a significantly lower incidence of cesarean delivery (38.8% vs 39.7%; RR: 0.69, 95% CIs: 0.53-0.91, *P*=0.008, *I*^*2*^=82%), compared with those who did not (Fig. 2). As for secondary outcomes, the use of anesthesia significantly reduced the incidence of vaginal breech delivery (0.5% vs 6.6%; RR: 0.16, 95% CIs: 0.04-0.74, *P*<0.001, *I*^*2*^=0%) but significantly increased the risk of hypotension (6.7% vs 0%; RR: 8.41, 95% CIs: 2.23-31.62,* P*<0.001, *I*^*2*^=0%) significantly. The use of anesthesia showed a significant decrease in maternal pain scores (SMD: -1.28, 95% CI: -1.96 to -0.61, *P*<0.001, *I*^*2*^=0%).

**Table 3:** Primary and secondary outcomes

**Study**	**Successful ECV**	**Vaginal delivery**	**Cesarean delivery**	**Vaginal breech delivery**	**Emergency Cesarean delivery**	**Transient fetal bradycardia**	**Nonreassuring fetal testing (excluding transient bradycardia) after ECV**	**Maternal discomfort**	**Maternal pain score**	**Abruption placentae**	**Vaginal bleeding or postpartum hemorrhage**	**Maternal satisfaction scores**	**Hypotension**
Schorr et al, 1997 [27]	24/35 (68.6%) vs 11/34 (32.3%)	23/35 (65.7%) vs 7/34 (20.6%)	12/35 (34.3%) vs 27/34 (79.4%)	Not reported	Not reported	Not reported	Not reported	1/35 (2.8%) vs 4/34 (11.8%)	Not reported	0/35(0%) vs 0/34(0%)	Not reported	Not reported	Not reported
Dugoff et al, 1999 [9]	22/50 (44.0%) vs 22/52 (42.3%)	16/50 (32.0%) vs 25/52 (48.1%)	34/50 (68.0%) vs 27/52 (51.9%	0/50 (0.0%) vs 0/52 (0.0%)	0/50 (0.0%) vs 1/52 (1.9%)	11/50 (22.0%) vs 6/52 (12.0%)	0/50 (0.0%) vs 1/52 (1.9%)	0/50 (0.0%) vs 4/52 (8.0%)	Not reported	0/50 (0.0%) vs 1/52 (1.9%)	Not reported	Not reported	4/50(8%) vs 0/52(0%)
Mancuso et al, 2000 [8]	32/54 (59.2%) vs 18/54(33.3%)	29/54 (53.7%) vs 17/54 (31.5%)	25/54 (46.3%) vs 37/54 (68.5%)	1/54 (1.8%) vs 3/54 (5.5%)	0/54 (0.0%) vs 0/54 (0.0%)	2/54 (3.7%) vs 3/54 (5.5%)	Not reported	Not reported	Not reported	0/54 (0.0%) vs 0/54 (0.0%)	Not reported	Not reported	0/54(0%) vs 0/54(0%)
Weiniger et al, 2007 [28]	24/36 (66.6%) vs 11/34 (32.3%)	Not reported	Not reported	Not reported	0/36 (0.0%) vs 0/34 (0.0%)	Not reported	2/36 (5.5%) vs 0/34 (0.0%)	Higher in control group	VAS (0=no pain, 10=severe pain) 1.76±2.7 vs 6.84±3.1	0/36 (0.0%) vs 0/34 (0.0%)	Not reported	Not reported	7/36(19.4%) vs 0/34(0%)
Weiniger et al, 2010 [29]	27/31 (87.0%) vs 19/33 (57.6%)	27/31 (87.1%) vs 30/33 (91.0%)	4/31 (12.9%) vs 3/33 (9.1%)	0/31 (0.0%) vs 3/33 (9.1%)	0/31 (0.0%) vs 0/33 (0.0%)	2/31 (6.4%) vs 1/33 (3.0%)	1/31 (3.0%) vs 0/33 (0.0%)	Higher in control group	VAS (0=*n*o pain, 10=severe pain) 1.7±2.4 vs 5.5±2.9	0/31 (0.0%) vs 0/33 (0.0%)	Not reported	Not reported	10/31(32%) vs 0/33(0%)
Burgos et al, 2013 [11]	157/300(52.3%) vs 79/150(52.7%)	Not reported	Not reported	Not reported	3/300(1%) vs 2/150(1.3%)	1/300(0.3%) vs 1/150(0.7%)	0/300(0.0%) vs 1/150(0.7%)	Not reported	Numeric rating scale (1–3: mild pain, 4–7: moderate pain, 8–10: severe pain) 6 [4–7] vs 7 [5–8]	Not reported	4/300(1.3%) vs 4/150(2.6%)	Not reported	Not reported
Munoz et al, 2014 [12]	17/31 (54.8%) vs 12/29 (41.3%)	14/31(45.2%) vs 11/29(37.9%)	17/31(54.8%) vs 16/29(55.2%)	Not reported	Not reported	3/31(9.7%) vs 9/29(31.0%)	Not reported	2/31(6.5%) vs 4/29(13.8%)	Numerical rating scale (0 = no pain, 10 = worst pain imaginable) 4.7 ± 2.5 vs 6.5 ± 2.4	Not reported	Not reported	Not reported	Not reported
Khaw et al, 2015 [10]	52/63 (82.5%) vs 40/63 (63.5%) vs 40/63(63.5%)	52/63(83%) vs 40/63(64%) vs 40/63(64%)	23/63(36.5%) vs 32/63(50.8%) vs 32/63(50.8%)	Not reported	3/63(4.8%) vs 6/63(9.6%) vs 3/63(4.8%)	Not reported	3/63(4.8%) vs 6/63(9.6%) vs 3/63(4.8%)	Higher in control group	VAS (0 mm=none, 100 mm=most extreme) 0[0-0]vs 35[0-60] vs 50 [30-75]	Not reported	Not reported	Not reported	Not reported
Liu et al, 2016 [14]	43/76(56.5%) vs 30/76(39.5%)	64/76(84.2%) vs 70/76(92.1%)	12/76(15.8%) vs 6/76(7.9%)	Not reported	Not reported	4/76(5.3%) vs 7/76(9.2%)	Not reported	Not reported	NRPS (0=no pain, 10=worst pain imaginable) 4.6±2.6 vs 6.5±2.7	Not reported	Not reported	Numerical rating scale (0=completely dissatisfied, 10=completely satisfied) 9.6 ±1.4 vs 6.4 ± 3.7	1/76(1.3%) vs 0/76(0%)
Wang et al, 2017 [30]	41/72 (56.9%) vs 28/72 (38.9%)	37/72(51.4%) vs 24/72(33.3%)	35/72(48.6%) vs 40/72(55.6%)	0/72(0%) vs 8/72(11.1%)	Not reported	2/72 (2.8%) vs 6/72 (8.3%)	Not reported	7/72(9.7%) vs 10/72(13.9%)	VAS scale (0 =no pain, 10= worst pain imaginable) 4.3±2.2 vs 6.4 ±2.5	Not reported	Not reported	Numerical rating scale (0 = completely dissatisfied, 10= completely satisfied) 9.3±0.9 vs 6.7±1.2	1/72 (1.4%) vs 0/72 (0%)
Yang et al, 2019 [31]	14/25 (56.0%) vs 7/21 (33.3%)	11/25 (44%) vs 5/21 (23.8%)	3/25(12%) vs 5/21(23.8%)	Not reported	Not reported	Not reported	2/25(8%) vs 1/21(4.8%)	0/25 (0.0%) vs 0/21 (0.0%)	VAS (0=no pain, 10=severe pain) 3.07±0.35 vs 6.47 ±0.51	0/25 (0.0%) vs 0/21 (0.0%)	2/25(8%) vs 2/21(9.5%)	Not reported	0/25 (0%) vs 0/21 (0%)
Dochez et al, 2020 [15]	18/74 (24.3%) vs 15/76 (19.7%)	36/74(48.6%) vs 28/76 (36.8%)	38/74 (51.4%) vs 47/76 (61.8%)	Not reported	17/74 (23.0%) vs 13/76 (17.1%)	Fetal bradycardia (*P* = 0.34; 95% CI 0.25 to 0.24)	Not reported	41/74 (55.4%) vs 7/76 (9.2%)	VAS scale (0 =no pain, 10= worst pain imaginable) 5.9 ± 2.4 vs 5.6 ± 2.3	Not reported	Not reported	A patient satisfaction questionnaire 87.5% vs 85.9%	Not reported
Zhang et al, 2020 [32]	28/40(70%) vs 13/40(32.5%)	Not reported	5/40(12.5%) vs 11/40(27.5%)	Not reported	Not reported	Not reported	Not reported	Not reported	Not reported	Not reported	Not reported	Not reported	Not reported
Han et al, 2020 [33]	46/63(69.8%) vs 13/41(31.8%)	54/63(85.7%) vs 21/41(51.2%)	9/63(14.3%) vs 20/41(48.8%)	Not reported	Not reported	Not reported	2/63 (3.1%) vs 3/41 (7.3%)	Not reported	Not reported	0/63 (0%) vs 1/41(2.4%)	2/63(3.2%) vs 2/41(4.9%)	Not reported	Not reported
Straube et al, 2021 [34]	8/23 (34.8%) vs 10/25 (40.0%)	Not reported	15/23 (65.2%) vs 17/25 (68.0%)	Not reported	0/23 (0.0%) vs 0/25 (0.0%)	0/23(0%) vs 2/25(8%)	0/23 (0.0%) vs 0/25 (0.0%)	8/23(34.8%) vs 4/25(16%)	A scale of 0–10 (0 = no pain, 10 = worst pain imaginable) 5.5 ± 2.3 vs 5.4 ± 2.7	0/23 (0.0%) vs 0/25 (0.0%)	Not reported	Using a standardized data collection sheet (0 = not at all satisfied, 10 = extremely satisfied) 4.3 ± 4.0 vs 6.9 ± 3.6	Not reported
Yang et al, 2023 [35]	44/67 (65.6%) vs 90/134 (67.2%)	Not reported	29/67 (43.3) vs 47/134 (35.1%)	Not reported	8/67 (11.9%) vs 6/134 (4.5%)	Not reported	Not reported	Not reported	Not reported	3/67 (4.5%) vs 0/134 (0%)	Not reported	Not reported	Not reported
Total	640/1103 (58.2%) vs 458/997 (45.9%)	403/637 (63.3%) vs 318/614 (51.8%)	298/767 (38.8%) vs 323/813 (39.7%)	1/207 (0.5%) vs 14/211 (6.6%)	37/561 (6.6%) vs 28/684 (4.1%)	24/314 (7.6%) vs 32/316 (10.1%)	16/654 (2.5%) vs 12/482 (2.5%)	59/310 (19.1%) vs 33/309 (10.7%)	-	3/384 (0.8%) vs 2/428 (0.5%)	8/388 (2.1%) vs 8/212 (3.8%)	-	23/344 (6.7%) vs 0/342 (0%)
*I*^*2*^(%)	59	74	82	0	0	38	0	79.3	94.1	0	0	97.2	0
RR or SMD	1.37(1.19-1.58)	1.23(1.03-1.47)	0.69(0.53-0.91)	0.16(0.04-0.74)	1.57(0.97-2.54)	0.72(0.36-1.47)	1.12(0.54-2.34)	0.96(0.30-3.02)	-1.28(-1.96 to -0.61)	1.67(0.28-9.92)	0.58(0.29-1.14)	0.98(-0.55-2.52)	8.41(2.23-31.62)

*ECV* External cephalic version, *RR* Relative risk, *SMD* Standardized mean difference, *VAS* Visual analog scale, *NRPS* Numerical Rating Pain Scale

However, there was no significant difference between groups for the incidences of emergency cesarean delivery (6.6% vs 4.1%; RR: 1.57, 95% CIs: 0.97-2.54, *P*=0.24, *I*^*2*^=0%), transient bradycardia (7.6% vs 10.1%; RR: 0.72, 95% CIs: 0.36-1.47, *P*=0.37, *I*^*2*^=38%), non-reassuring fetal testing, excluding transient bradycardia, after ECV (2.5% vs 2.5%; RR: 1.12, 95% CIs: 0.54-2.34, *P*=0.36, *I*^*2*^=0%), maternal discomfort (19.1% vs 10.7%; RR: 0.96, 95% CIs: 0.30-3.02, *P*=0.28, *I*^*2*^=79.3%), abruption placentae (0.8% vs 0.5%; RR: 1.67, 95% CIs: 0.28-9.92, *P*=0.26,* I*^*2*^=0%), vaginal bleeding or postpartum hemorrhage (2.1% vs 3.8%; RR: 0.58, 95% CIs: 0.29-1.14, *P*=0.54, *I*^*2*^=0%) and maternal satisfaction scores (SMD: 0.98, 95% CIs: -0.55 to 2.52, *P*=0.42, *I*^*2*^=97.2%).

Tables 4 and 5 shows the results of the subgroup analysis according to the different types of anesthesia and regions.

**Table 4:** Subgroup analysis of successful ECV, vaginal delivery and cesarean delivery among different types of anesthesia

**Outcomes**	**Number of studies**	***I***^***2***^**(%)**	**RRs**	**95%CIs**	***P*-value**
*Successful ECV*					
General anesthesia	8	19.9	1.16	1.00-1.34	0.052
Inhalation anesthesia	3	0	1.00	0.84-1.19	0.244
Intravenous anesthesia	5	17.6	**1.27**	**1.05-1.54**	0.005
Regional anesthesia	9	37.8	**1.65**	**1.38-1.98**	<0.001
Spinal anesthesia	7	71.0	**1.48**	**1.17-1.89**	0.003
Epidural anesthesia	2	0	**1.91**	**1.36-2.68**	0.005
Total	17	59.0	**1.37**	**1.19-1.58**	<0.001
*Vaginal delivery*					
General anesthesia	6	54.9	1.15	0.95-1.40	0.072
Inhalation anesthesia	1	NA	1.32	0.91-1.92	0.083
Intravenous anesthesia	5	54.3	1.10	0.88-1.38	0.261
Regional anesthesia	6	81.0	1.32	0.98-1.80	0.062
Spinal anesthesia	4	80.5	1.12	0.83-1.52	0.362
Epidural anesthesia	2	53.0	**2.20**	**1.20-4.03**	<0.001
Total	12	74.0	**1.23**	**1.03-1.47**	<0.001
*Cesarean delivery*					
General anesthesia	7	25.9	0.92	0.77-1.11	0.227
Inhalation anesthesia	2	0	0.87	0.69-1.10	0.327
Intravenous anesthesia	5	44.2	1.02	0.76-1.38	0.242
Regional anesthesia	8	76.8	**0.65**	**0.43-0.97**	0.005
Spinal anesthesia	6	88.0	0.62	0.34-1.14	0.12
Epidural anesthesia	2	53.9	**0.56**	**0.36-0.86**	<0.001
Total	15	82.0	**0.69**	**0.53-0.91**	0.008

*ECV* External cephalic version, *RR* Relative risk, *CIs* Confidence intervals

**Table 5:** Subgroup analysis of successful ECV in different regions

**Subgroup**	**Number of studies**	***I***^***2***^**(%)**	**RRs**	**95%CIs**	***P*-value**
Asia	10	72.1	**1.41**	**1.12-1.68**	0.005
Europe	3	0	1.04	0.88-1.23	0.323
North America	4	45.4	**1.46**	**1.03-2.07**	<0.001

*ECV* External cephalic version, *RR* Relative risk, *CIs* Confidence intervals

Subgroup analysis of different anesthesia types (Table 4) showed that regional anesthesia significantly increased the success rate of ECV. The ECV success rate with regional anesthesia was 1.65 times higher than that without anesthesia (RR: 1.65, 95% CIs: 1.38-1.98, *P*<0.001, *I*^*2*^=37.8%), whereas the use of general anesthesia did not have a significant effect on ECV success (RR: 1.16, 95% CIs: 1.00-1.34, *P*=0.052, *I*^*2*^= 19.9%). Comparison of the different types of regional anesthesia revealed that both spinal anesthesia (RR: 1.48, 95% CIs: 1.17-1.89, *P*=0.003, *I*^*2*^=71.0%) and epidural anesthesia (RR: 1.91, 95% CIs: 1.36-2.68, *P*=0.005,* I*^*2*^=0%) significantly increased the success rate of ECVs, with the use of epidural anesthesia had a greater likelihood of ECV success. For the different types of general anesthesia, inhalation anesthesia (RR: 1.00, 95% CIs: 0.84-1.19, *P*=0.244, *I*^*2*^ =0%) did not have a significant effect on ECV success, whereas intravenous anesthesia (RR: 1.27, 95% CIs: 1.05-1.54, *P*=0.005, *I*^*2*^=17.6%) significantly improved ECV success. Moreover, we found that regional anesthesia significantly reduced the incidence of cesarean delivery (RR: 0.65, 95% CIs: 0.43-0.97, *P*=0.005, *I*^*2*^=76.8%), while there was no statistically significant correlation between general anesthesia (RR: 0.92, CI: 0.77-1.11, *P*=0.227, *I*^*2*^=25.9%) and the incidence of cesarean delivery. In terms of vaginal delivery rate, we found that although regional anesthesia (RR: 1.32, CI: 0.98-1.80, *P*=0.062, *I*^*2*^=81.0%) had no significant effect on vaginal delivery rate, one type of regional anesthesia, epidural anesthesia (RR: 2.20, CIs: 1.20-4.03, *P*<0.001, *I*^*2*^=53.0%), significantly increased the vaginal delivery rate. Neither general anesthesia nor the specific type (inhalation anesthesia, intravenous anesthesia) was significantly associated with vaginal delivery rates. The above results showed that there are differences in the effects of different types of anesthesia on the success rate of ECV, the incidence of vaginal delivery, and the incidence of cesarean delivery; however, there is a large overlap in the 95% CIs of the effect values for the different subgroups, and therefore the differences between the subgroups cannot yet be considered statistically significant.

For the different regions (Table 5), we extracted raw data from 17 studies (Khaw et al., 2015 [10] included two RCTs). The results showed that anesthesia significantly increased the success rate of the ECV in Asia (RR: 1.41, 95% CIs: 1.12-1.68, *P*=0.005, *I*^*2*^=72.1%) and North America (RR: 1.46, 95% CIs: 1.03-2.07, *P*<0.001, *I*^*2*^=45.4%). In Europe, anesthesia improved the success rate of the ECV, but the difference was not statistically significant (RR: 1.04, 95% CIs: 0.88-1.23, *P* = 0.323, *I*^*2*^ = 0%). The above results showed that there are differences in the effects of different regions on the success rate of ECV; however, there is a large overlap in the 95% CIs of the effect values for the different subgroups, and therefore the differences between the subgroups cannot yet be considered statistically significant.

### Study quality assessment and risk of *bias*

**Fig. 3:**
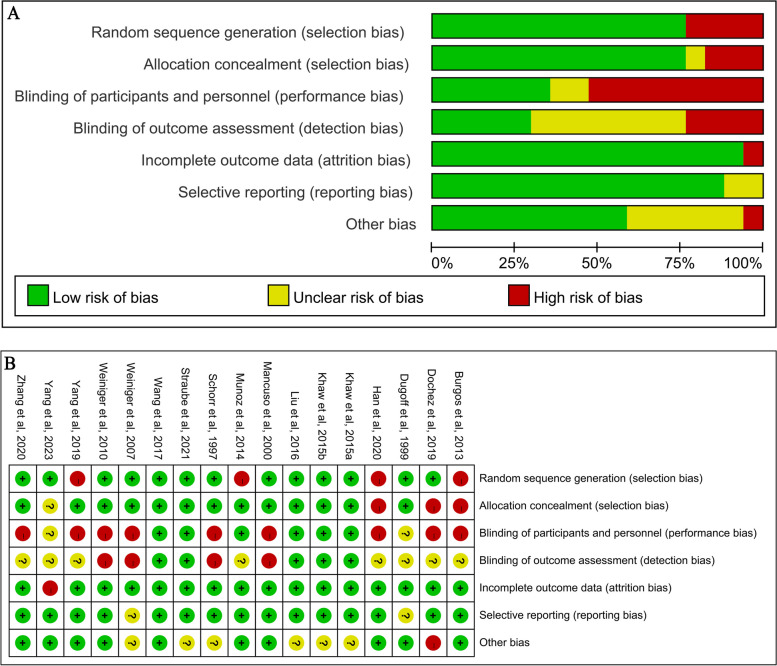
Risk of bias graph (**A**) and summary (**B**)

**Fig. 4:**
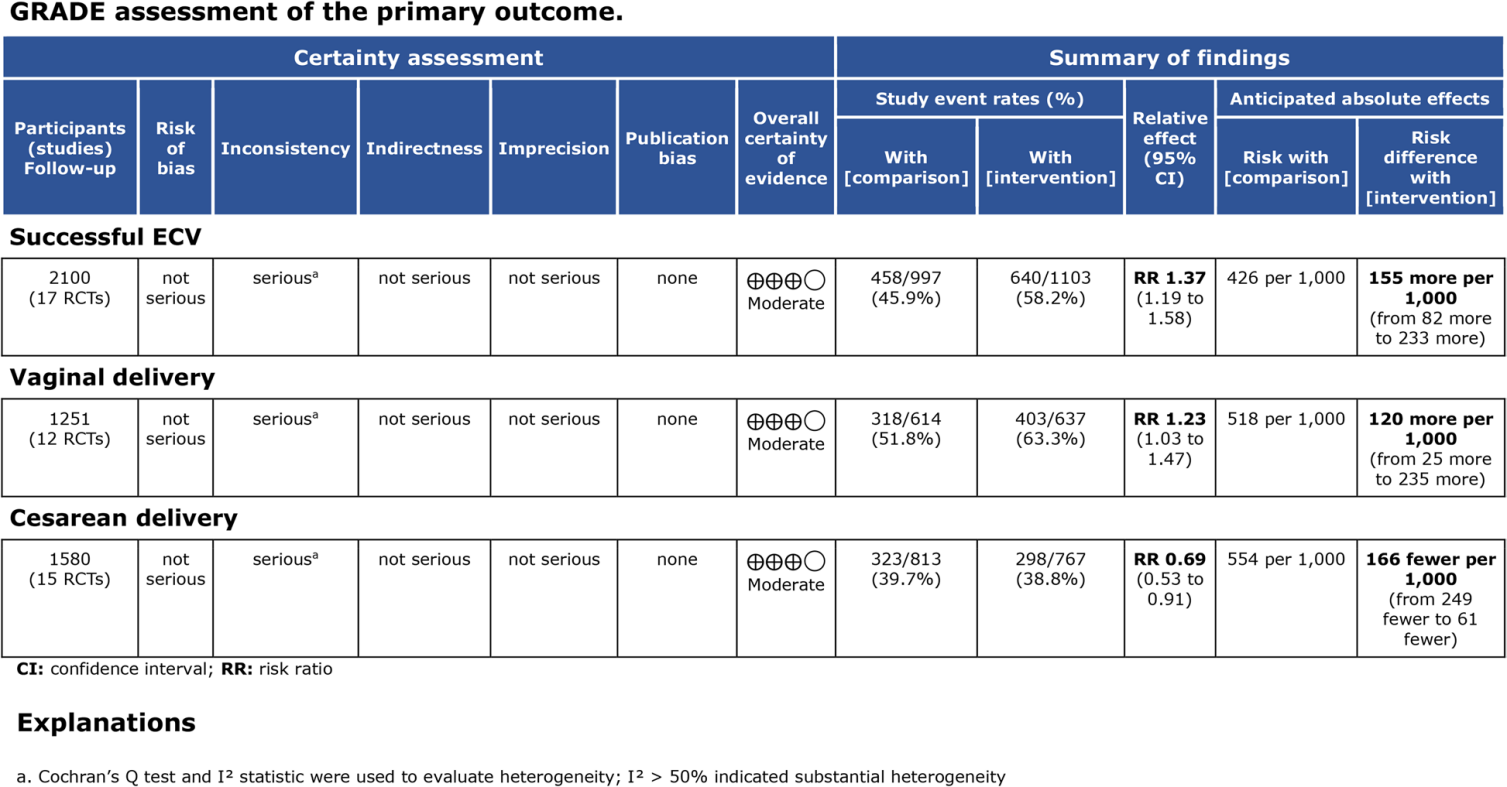
GRADE assessment of the primary outcomes

**Fig. 5:**
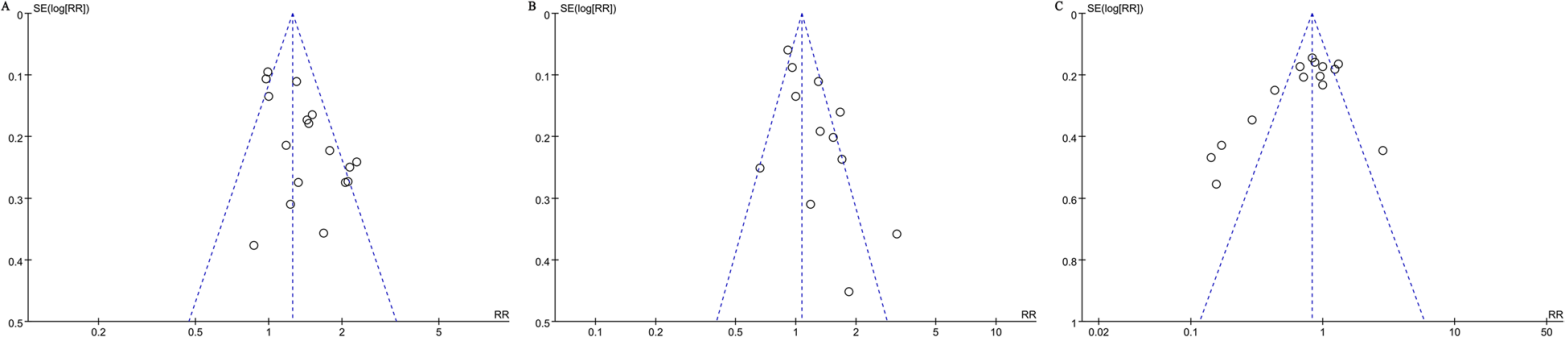
Funnel plots for successful ECV (**A**), vaginal delivery (**B**), cesarean delivery (**C**)

Using the Cochrane risk of bias assessment tool, all RCTs included in the meta-analysis had low risks of bias in most criteria. The risk of bias graph and summary are illustrated in Fig. 3. The five domains where most assessments were ‘low’ risk were “random sequence generation” (*n*=13), “allocation concealment” (*n*=13), “incomplete outcome data” (*n*=16), “selective reporting” (*n*=15) and “other bias” (*n*=10). ‘Unclear’ risk assessments were most common in “blinding of participants and personnel” (*n*=8). Only one domain with the most ‘high’ risk assessments were “blinding of participants and personnel” (*n*=9). The study with the least risk of bias assessments was Wang et al. [31] with seven low risk assessments. Four studies (two are included in Khaw et al, 2015) had six ‘low’ risk assessments across domains [10, 14, 35]. Two studies with the most (n=3) uncertainty in risk of bias across domains was Yang et al. [32] and Dugoff et al. [9]. The studies with the highest risk of bias across domains (n=3) were Han et al. [34], Dochez et al. [15] and Burgos et al. [11]. Most of the included studies showed an overall low risk of bias. However, for domains 4 and 5: *Blinding of participants and personnel* and *Blinding of outcome assessment*, the overall risk of bias was high. The fact that most studies did not blind medical personnel and that anesthetic injections and surgical procedures were performed by them contributed to the high risk of this bias. Because the behavioral intervention process cannot be fully blinded, this bias is present in all studies and ultimately leads to an overestimation of effects. There was a low risk of bias in the Cochrane risk of bias assessment, and the GRADE assessment showed a moderate to high certainty of anesthesia's effect on the primary outcomes (Fig. 4). In addition, the funnel plot, constructed from the studies corresponding to successful ECV, vaginal delivery and cesarean delivery, did not suggest the presence of potential publication bias (Fig. 5). The Begg's test and Egger's test did not reveal publication bias. In this study, for successful ECV, the *P*-value for Begg's test was 0.149 and for Egger's test was 0.005. For vaginal delivery, the *P*-value for Begg's test was 0.115 and for Egger's test was 0.013. For cesarean delivery, the *P*-value for Begg's test was 0.322 and for Egger's test was 0.502.

### Sensitivity analysis

**Fig. 6:**
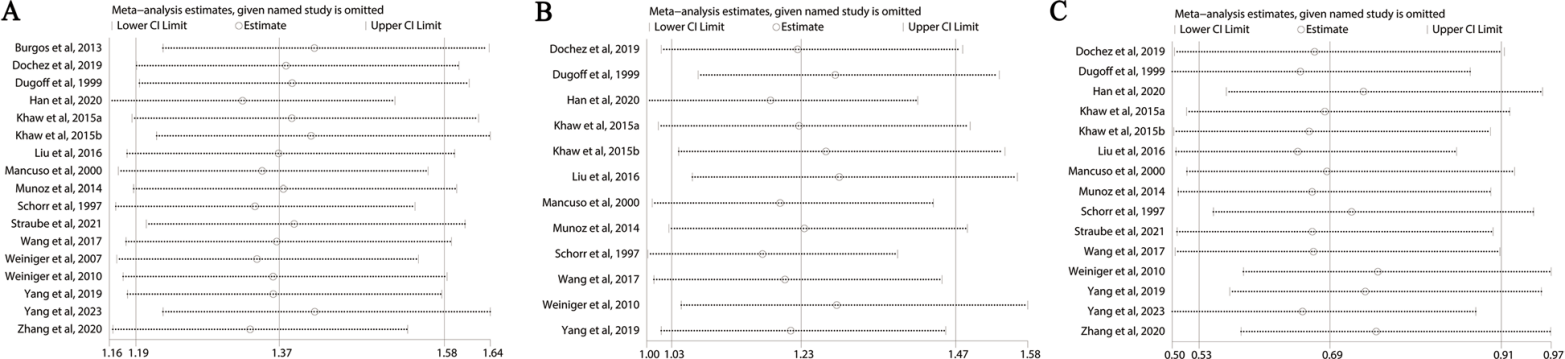
Sensitivity analysis graph of anesthesia with successful ECV (**A**), vaginal delivery (**B**), and cesarean delivery (**C**)

For successful ECV, vaginal delivery, and cesarean delivery, the conclusions were not altered after excluding each article to calculate the heterogeneity and effect size. Sensitivity analysis results are shown in Fig. 6.

## Discussion

It is a widely recognized fact that the primary objective of ECV is to reposition the fetus in order to increase the likelihood of vaginal delivery. Our meta-analysis indicates that the success rate of ECV among pregnant women who were anesthetized was 1.37 times higher than those who were not anesthetized. Moreover, the rate of vaginal delivery was 1.23 times higher in the anesthetized group, and the risk of cesarean delivery was significantly lower. However, it should be noted that not all types of anesthesia were equally effective. Inhalation anesthesia did not have a statistically significant effect on the success rate of ECV compared to the control group without anesthesia. Our meta-analysis included three studies that employed nitrous oxide (N_2_O) as an analgesic gas for inhalation anesthesia, but none of them showed a significant effect on the success rate of ECV [11, 15, 35]. N_2_O is an easy-to-use and safe analgesic, but its analgesic power is only one-third to one-half that of clinical doses of intravenous fentanyl [37], which may not be sufficient to affect the performance of ECV procedures. Intravenous, spinal, and epidural anesthesia have all been found to contribute to successful ECV. However, it was observed that only the administration of epidural anesthesia led to a significant increase in the rate of vaginal delivery and a decrease in the rate of cesarean delivery. The role of epidural anesthesia was also supported by Mancuso et al., who reported a 1.8-fold increase in ECV success and a 2.2-fold increase in vaginal delivery of cephalic infants under epidural anesthesia [8]. Conversely, spinal anesthesia, which is another form of regional anesthesia, did not have a significant effect on the rate of cesarean delivery or vaginal delivery. This may be due to the differences in characteristics of the anesthesia and the mechanism of action. In conclusion, our findings suggest that epidural anesthesia may be more suitable for ECV, and that further research should explore the specific types and doses of anesthetics required for optimal epidural anesthesia.

Although the utilization of intravenous and spinal anesthesia increased the success rate of ECV, its effectiveness on vaginal delivery or cesarean delivery did not been significantly demonstrated. The use of anesthesia during ECV may have implications for cesarean and vaginal delivery. Specifically, the expected discomfort and pain during ECV might prompt some pregnant women to decline the procedure, choosing instead to have a cesarean section during childbirth. And the use of anesthesia may increase their willingness to undergo ECV and improve the success of the procedure while increasing the likelihood of vaginal delivery [36, 38]. However, there are many other factors that affect cesarean and vaginal delivery. One study conducted on pregnant women in Thailand discovered that a history of vaginal delivery, maternal body mass index (BMI), estimated fetal weight and gestational age were all significant factors affecting the probability of a successful vaginal delivery [39]. In many cases, the decision to perform a cesarean section is often highly dependent on the obstetrician-gynecologist. Consequently, for fetuses in BP, the healthcare providers should make an evaluation based on multiple factors, taking into account not only the appropriate type of anesthesia for ECV, but also the necessity of performing ECV.

Furthermore, we observed a minimal occurrence of adverse events in all studies; however, the use of anesthesia may heighten the possibility of maternal hypotension. Despite the wide CIs in the meta-analysis regarding hypertension, possibly influenced by heterogeneity, our results indicate that the potential harm caused by anesthesia warrants significant attention in clinical practice. We speculated that the heterogeneity might stem from variations in inclusion and exclusion criteria across the studies or differences in the methods used to measure blood pressure. Only the study by Mancuso et al. [8] provided details on the measurement of maternal blood pressure, while no such information was reported in the other studies. Maternal hypotension is a common complication of spinal anesthesia and has a strong correlation with perinatal outcomes [40]. This is primarily because sympathetic relaxation produced by spinal blockade leads to vasodilation, which can result in maternal hypotension. Maternal hypotension may impact uterine blood flow and fetal circulation, leading to fetal hypoxia, bradycardia, and acidosis [41]. In the event that anesthesia is employed during ECV surgery, it is crucial to monitor any changes in maternal blood pressure and implement preventive and corrective measures promptly.

Our study discovered distinct impacts of anesthesia use on the effectiveness of ECV in various regions, which may be attributed to the primary type of anesthesia employed, the ECV surgical technique, and cultural disparities in pain management. Regrettably, the limited quantity of relevant studies prevented us from conducting a more detailed analysis by further categorizing the type of anesthesia. Such categorization can be conducted in subsequent studies to refine the findings.

The strengths of our study are the inclusion of relevant RCTs published to date, the low bias of included studies, and the moderate quality. At the same time, there are some limitations of this study. First, although our meta-analysis performed subgroup analyses of different types of anesthesia, the anesthetic drugs used in different studies and the doses of the drugs were different, which may have an impact on the results. Currently, there is a lack of uniform standards internationally regarding anesthesia care for ECV, and the further studies of anesthetic drugs could be conducted to try to explore the types of anesthetic drugs and doses used that are most appropriate for ECV. Second, there was large overlap in the 95% CIs of effects between groups in the subgroup analysis results of this study. Although one subgroup analysis was statistically significant while another was not, the latter may simply reflect a lack of information rather than a smaller (or no) effect [42]. Thus, our study was only able to explore possible sources of heterogeneity through subgroup analyses and could not clarify differences between subgroups. Third, maternal age, parity, and estimated fetal weight may be confounding factors in different studies [43], and the heterogeneity caused by these factors is difficult to control. Finally, most of the RCTs we included were designed to study successful ECV as a primary outcome and were not sufficiently competent to detect differences in the incidence of cesarean delivery and other perinatal outcomes, resulting in our meta-analysis of other outcomes being less comprehensive.

## Conclusion

The administration of anesthesia not only significantly reduces maternal pain, but also significantly increases the success rate of ECV among women with malpresentation at term, which then increases the incidence of vaginal delivery and reduces the incidence of cesarean delivery. However, it may increase the incidence of maternal hypotension. Possible confounders such as maternal age, parity, estimated fetal weight, etc. need to be controlled for to further refine the meta-analysis.

### Supplementary Information

Additional file 1: Table s1.Search strategy used on January 2, 2024.

Additional file 2: Table s2. Scales and time points for outcomes of studies.

## Data Availability

The datasets used and/or analysed during the current study are available from the corresponding author on reasonable request.
