# Supplementary material for: Effect of anesthesia on the success rate of external cephalic version: GRADE- assessed systematic review and meta-analysis of randomized controlled trials
Source: Syst Rev. 2024 Jul 30;13:202. doi: 10.1186/s13643-024-02616-y (PMC11290114; doi:10.1186/s13643-024-02616-y)
Supplement: Supplementary file 1 — Additional file 1: Table s1.Search strategy used on January 2, 2024. [file 13643_2024_2616_MOESM1_ESM.docx]

**Table s1 Search strategy used on January 2, 2024**

| **Database** |  | **Query** | **Items found** |
| --- | --- | --- | --- |
| Web of Science  208 | 1 | TS= ("Version, fetal" OR "Breech presentation” OR "External cephalic version") | 5,565 |
|  | 2 | TS=(“Anesthesia neuraxial” OR “Anesthesia, epidural” OR “Anesthesia, spinal” OR “Anesthesia, obstetrical” OR “Anesthesia, local” OR “Regional anesthesia” OR “Anesthesia, general” OR “Anesthesia, endotracheal” OR “Balanced anesthesia” OR “Anesthesia and analgesia” OR “Anesthetic interventions” OR “Anesthesia, inhalation” OR “Nitrous Oxide” OR “Sevoflurane” OR “Halothane” OR “Anesthesia, injections” OR “Anesthesia, intravenous” OR “Hypnotics and sedatives” OR “Narcotics” OR “Opioid” OR “Morphine” OR “Fentanyl” OR “Propofol” OR “Dexmedetomidine” ) | 489,295 |
|  | 3 | #1 AND #2 | 220 |
|  | 4 | Limit 3 to “Humans” | 208 |
| PubMed  10 | 1 | "Version, fetal"[Title/Abstract] OR "Breech presentation"[Title/Abstract] OR "External cephalic version"[Title/Abstract] OR "Version, Fetal"[Mesh] | 3,532 |
|  | 2 | "Anesthesia neuraxial"[Title/Abstract] OR "Anesthesia, epidural"[Title/Abstract] OR "Anesthesia, spinal"[Title/Abstract] OR "Anesthesia, obstetrical"[Title/Abstract] OR "Anesthesia, local"[Title/Abstract] OR "regional anesthesia"[Title/Abstract] OR "Anesthesia, general"[Title/Abstract] OR "Anesthesia, endotracheal"[Title/Abstract] OR "Balanced Anesthesia"[Title/Abstract] OR "Anesthesia and analgesia"[Title/Abstract] OR "anesthetic interventions"[Title/Abstract] OR "Anesthesia, inhalation"[Title/Abstract] OR "Nitrous Oxide"[Title/Abstract] OR "Sevoflurane"[Title/Abstract] OR "Halothane"[Title/Abstract] OR "anesthesia, injections"[Title/Abstract] OR "Anesthesia, intravenous"[Title/Abstract] OR "Hypnotics and sedatives"[Title/Abstract] OR "Narcotics"[Title/Abstract] OR "Opioid"[Title/Abstract] OR "Morphine"[Title/Abstract] OR "Fentanyl"[Title/Abstract] OR "Propofol"[Title/Abstract] OR "Dexmedetomidine"[Title/Abstract] OR "Anesthesia/therapy"[Mesh] | 237,762 |
|  | 3 | #1 AND #2 | 52 |
|  | 4 | Filters: Randomized Controlled Trial | 10 |
| Embase  66 | 1 | 'version, fetal' OR 'breech presentation' OR 'external cephalic version' | 7,424 |
|  | 2 | 'anesthesia neuraxial' OR 'anesthesia, epidural' OR 'anesthesia, spinal' OR 'anesthesia, obstetrical' OR 'anesthesia, local' OR 'regional anesthesia' OR 'anesthesia, general' OR 'anesthesia, endotracheal' OR 'balanced anesthesia' OR 'anesthesia and analgesia' OR 'anesthetic interventions' OR 'anesthesia, inhalation' OR 'nitrous oxide' OR 'sevoflurane' OR 'halothane' OR 'anesthesia, injections' OR 'anesthesia, intravenous' OR 'hypnotics and sedatives' OR 'narcotics' OR 'opioid' OR 'morphine' OR 'fentanyl' OR 'propofol' OR 'dexmedetomidine' | 1,774,310 |
|  | 3 | #1 AND #2 | 908 |
|  | 4 | #3 AND ('randomized controlled trial'/de OR 'randomized controlled trial topic'/de) | 66 |
| Cochrane  53 | 1 | (“Version, fetal” OR “Breech presentation” OR “External cephalic version”): ti,ab,kw | 426 |
|  | 2 | (“Anesthesia neuraxial” OR “Anesthesia, epidural” OR “Anesthesia, spinal” OR “Anesthesia, obstetrical” OR “Anesthesia, local” OR “regional anesthesia” OR “Anesthesia, general” OR “Anesthesia, endotracheal” OR “Balanced Anesthesia” OR “Anesthesia and analgesia” OR “anesthetic interventions” OR “Anesthesia, inhalation” OR “Nitrous Oxide” OR “Sevoflurane” OR “Halothane” OR “anesthesia, injections” OR “Anesthesia, intravenous” OR “Hypnotics and sedatives” OR “Narcotics” OR “Opioid” OR “Morphine” OR “Fentanyl” OR “Propofol” OR “Dexmedetomidine”):ti,ab,kw | 90,078 |
|  | 3 | #1 AND #2 | 53 |
| Chinese databases  82 | 1 | “Version, fetal” OR “Breech presentation” OR “External cephalic version” (used corresponding Chinese) |  |
|  | 2 | “Anesthesia neuraxial” OR “Anesthesia, epidural” OR “Anesthesia, spinal” OR “Anesthesia, obstetrical” OR “Anesthesia, local” OR “regional anesthesia” OR “Anesthesia, general” OR “Anesthesia, endotracheal” OR “Balanced Anesthesia” OR “Anesthesia and analgesia” OR “anesthetic interventions” OR “Anesthesia, inhalation” OR “Nitrous Oxide” OR “Sevoflurane” OR “Halothane” OR “anesthesia, injections” OR “Anesthesia, intravenous” OR “Hypnotics and sedatives” OR “Narcotics” OR “Opioid” OR “Morphine” OR “Fentanyl” OR “Propofol” OR “Dexmedetomidine”(used corresponding Chinese) |  |
|  | 3 | #1 AND #2 | 82 |
