# Supplementary material for: Effect of anesthesia on the success rate of external cephalic version: GRADE- assessed systematic review and meta-analysis of randomized controlled trials
Source: Syst Rev. 2024 Jul 30;13:202. doi: 10.1186/s13643-024-02616-y (PMC11290114; doi:10.1186/s13643-024-02616-y)
Supplement: Supplementary file 2 — Additional file 2: Table s2. Scales and time points for outcomes of studies. [file 13643_2024_2616_MOESM2_ESM.docx]

| **Table s2. Scales and time points for outcomes of studies** | | | | | |
| --- | --- | --- | --- | --- | --- |
| **Study** | **Time points** | **Primary outcomes** | **Scales ^a^** | **Secondary outcomes** | **Scales ^b^** |
| Schorr et al, 1997 [27] | From 1 December 1993 to 31 July 1996 | Successful ECV; Incidence of vaginal delivery; Incidence of cesarean delivery | Successful ECV: Success was defined by ultrasonographic visualization of a fetal vertex presentation. Incidence of vaginal delivery: Ratio of vaginal births to total deliveries. Incidence of cesarean delivery: Ratio of caesarean section deliveries to total deliveries. | Maternal discomfort | Maternal discomfort: Ratio of number of discomforted mothers to total number of mothers. |
| Dugoff et al, 1999 [9] | Between October 1993 and August 1997 | Successful ECV; Incidence of vaginal delivery; Incidence of cesarean delivery | Successful ECV: Real-time ultrasonography was used between attempts to assess fetal heart rate (FHR) and position. Incidence of vaginal delivery: Ratio of vaginal births to total deliveries. Incidence of cesarean delivery: Ratio of caesarean section deliveries to total deliveries. | Transient bradycardia, hypotension, abruption placentae, maternal discomfort. | Transient bradycardia: NA Hypotension: NA Abruption placentae: NA Maternal discomfort: Ratio of number of discomforted mothers to total number of mothers. |
| Mancuso et al, 2000 [8] | From December 1, 1994, until June 30, 1998 | Successful ECV; Incidence of vaginal delivery; Incidence of cesarean delivery | Successful ECV: NA Incidence of vaginal delivery: Ratio of vaginal births to total deliveries. Incidence of cesarean delivery: Ratio of caesarean section deliveries to total deliveries. | Transient bradycardia, incidence of vaginal breech delivery | Transient bradycardia: NA Incidence of vaginal breech delivery: Ratio of vaginal breech births to total births. |
| Weiniger et al, 2007 [28] | From September 2002 to May 2006 | Successful ECV | Successful ECV: This was ascertained by ultrasonography and confirmed by both of the two obstetricians performing the external cephalic version. | VAS pain score, hypotension | VAS pain score: 0 no pain, 10 severe pain. Hypotension: greater than 20-mm Hg reduction in systolic blood pressure. |
| Weiniger et al, 2010 [29] | January 20–25, 2002 | Successful ECV; Incidence of vaginal delivery; Incidence of cesarean delivery | Successful ECV: The primary endpoint was successful ECV confirmed using ultrasound. Incidence of vaginal delivery: Ratio of vaginal births to total deliveries. Incidence of cesarean delivery: Ratio of caesarean section deliveries to total deliveries. | Transient bradycardia, VAS pain score, hypotension. | Transient bradycardia: NA VAS pain score: 0, no pain; 10, severe pain. Hypotension: >20% reduction in systolic arterial pressure. |
| Burgos et al, 2013 [11] | September 2009 to December 2010 | Successful ECV; Incidence of cesarean delivery | Successful ECV: We considered the ECV successful when a breech presentation was converted to a cephalic presentation.  Incidence of cesarean delivery: Ratio of caesarean section deliveries to total deliveries. | Emergency cesarean section rate, pain score after the ECV. | Emergency cesarean section rate: Ratio of the number of emergency caesarean sections to the total number of caesarean sections performed. Pain score after the ECV: A pain score of 1–3 was categorized as mild pain, 4–7 as moderate pain, and 8–10 as severe pain. |
| Munoz et al, 2014 [12] | April 2010–March 2011 | Successful ECV; Incidence of vaginal delivery; Incidence of cesarean delivery | Successful ECV: NA Incidence of vaginal delivery: Ratio of vaginal births to total deliveries. Incidence of cesarean delivery: Ratio of caesarean section deliveries to total deliveries. | Maternal pain, satisfaction score, transient bradycardia, pain score. | Maternal pain: Ratio of number of discomforted mothers to total number of mothers. Satisfaction score: 0 = completely dissatisfied, 10 = completely satisfied. Transient bradycardia: NA Pain score: 0 = no pain, 10 = worst pain imaginable. |
| Khaw et al, 2015 [10] | From 1 May 2003 to 1 October 2008 | Successful ECV; Incidence of vaginal delivery; Incidence of cesarean delivery | Successful ECV: NA Incidence of vaginal delivery: Ratio of vaginal births to total deliveries. Incidence of cesarean delivery: Ratio of caesarean section deliveries to total deliveries. | Visual Pain Score, Visual Sedation Score, emergency cesarean, placental abruption | Visual Pain Score: 0 mm=none, 100 mm=most extreme Visual Sedation Score: 0 mm=none, 100 mm=most extreme Emergency cesarean: Ratio of number of discomforted mothers to total number of mothers Placental abruption: NA |
| Liu et al, 2016 [14] | Between January 2012 and December 2015 | Successful ECV; Incidence of vaginal delivery; Incidence of cesarean delivery | Successful ECV: NA Incidence of vaginal delivery: Ratio of vaginal births to total deliveries. Incidence of cesarean delivery: Ratio of caesarean section deliveries to total deliveries. | Maternal pain, transient bradycardia, Numerical Rating Pain Scale (NRPS) score, satisfaction score. | Maternal pain: Ratio of number of discomforted mothers to total number of mothers. Transient bradycardia: NA Pain score: 0=no pain, 10=worst pain imaginable Satisfaction score: 0=completely dissatisfied, 10=completely satisfied |
| Wang et al, 2017 [30] | From May 2013 to April 2016 | Successful ECV; Incidence of vaginal delivery; Incidence of cesarean delivery | Successful ECV: NA Incidence of vaginal delivery: Ratio of vaginal births to total deliveries. Incidence of cesarean delivery: Ratio of caesarean section deliveries to total deliveries. | Maternal pain, transient bradycardia, VAS pain score, satisfaction score. | Maternal pain: Ratio of number of discomforted mothers to total number of mothers. Transient bradycardia: NA Pain score: 0=no pain, 10=worst pain imaginable Satisfaction score: 0=completely dissatisfied, 10=completely satisfied |
| Yang et al, 2019 [31] | From January 2015 to March 2019 | Successful ECV; Incidence of vaginal delivery; Incidence of cesarean delivery | Successful ECV: Two doctors working together, gentle rotation, ultrasound confirms successful rotation to cephalic position. Incidence of vaginal delivery: Ratio of vaginal births to total deliveries. Incidence of cesarean delivery: Ratio of caesarean section deliveries to total deliveries. | Placental abruption, postpartum hemorrhage. | Placental abruption: NA Postpartum hemorrhage: NA |
| Dochez et al, 2020 [15] | From November 2013 through November 2015 | Successful ECV; Incidence of vaginal delivery; Incidence of cesarean delivery | Successful ECV: Success was defined by ultrasonographic visualization of a fetal vertex presentation. Incidence of vaginal delivery: Ratio of vaginal births to total deliveries. Incidence of cesarean delivery: Ratio of caesarean section deliveries to total deliveries. | Transient bradycardia, degree of pain with VAS, emergency cesarean. | Transient bradycardia: NA Degree of pain with VAS: from 0 for no pain to 10 for the maximum imaginable, with an unmarked 100-mm line. Emergency cesarean: Ratio of number of discomforted mothers to total number of mothers. |
| Zhang et al, 2020 [32] | From January 2019 to January 2020 | Successful ECV; Incidence of cesarean delivery | Successful ECV: NA Incidence of cesarean delivery: Ratio of caesarean section deliveries to total deliveries. | None | None |
| Han et al, 2020 [33] | From January 2017 to June 2019 | Successful ECV; Incidence of vaginal delivery; Incidence of cesarean delivery | Successful ECV: Success was defined by ultrasonographic visualization of a fetal vertex presentation. Incidence of vaginal delivery: Ratio of vaginal births to total deliveries. Incidence of cesarean delivery: Ratio of caesarean section deliveries to total deliveries. | Postpartum hemorrhage, placenta abruption, maternal discomfort. | Postpartum hemorrhage: NA Placenta abruption: NA Maternal discomfort: Ratio of number of discomforted mothers to total number of mothers. |
| Straube et al, 2021 [34] | From January 2017 to February 2019 | Successful ECV; Incidence of cesarean delivery | Successful ECV: NA Incidence of cesarean delivery: Ratio of caesarean section deliveries to total deliveries. | Maternal pain, and satisfaction. | Maternal pain: 0 = no pain, 10 = worst pain imaginable. Satisfaction score: 0 = not at all satisfied, 10 = extremely satisfied |
| Yang et al, 2023 [35] | From December 1st 2014 to December 31st 2022 | Successful ECV | Successful ECV: Success was defined by ultrasonographic visualization of a fetal vertex presentation. | Placenta abruption, emergency cesarean, and hemorrhage. | Placenta abruption: NA Hemorrhage: NA Emergency cesarean: Ratio of number of discomforted mothers to total number of mothers. |
| Notes: ECV, external cephalic version; VAS, visual analog scale; NA, No available; a, Scales for primary outcomes; b, Scales for secondary outcomes. | | | | | |
